# Barriers and Facilitators Towards Physiotherapists' Use of Behaviour Change Techniques (BCTs) to Improve Patients' Adherence to Treatment

**DOI:** 10.1111/jep.70339

**Published:** 2025-12-15

**Authors:** John Greenwood, Faith Alexandra Dohutimi Johnson, Christopher McCarthy, Nicola Middlebrook, Jasmine Heath Hearn

**Affiliations:** ^1^ Manchester Metropolitan University Manchester UK

**Keywords:** behaviour change, chronic pain, health psychology, qualitative

## Abstract

**Purpose:**

Patient adherence to physiotherapy treatment recommendations is less than optimal. This can lead to poor outcomes across a range of clinical and service level measures including patient satisfaction, long‐term morbidity and increased prevalence of long‐term conditions. Physiotherapists have been found to only use a small number of behaviour change techniques (BCTs) in consultations, despite evidence that these can help to improve outcomes in a range of domains including adherence. The study aimed to explore physiotherapists perceived barriers and facilitators to utilising BCTs in clinical practice.

**Methods:**

Fourteen qualified physiotherapists recruited from a higher education institution and a national healthcare provider participated in semi‐structured interviews and reflexive thematic analysis was conducted thereafter.

**Results:**

Three key themes were identified: (1) A Lack of Training and Support; (2) Organisational Culture and Practices; (3) Individual Differences During the Consultation, which had two subthemes: (i) Patient Attitudes and Expectations; (ii) Clinician Attitudes and Approach. Findings suggest that physiotherapists believe that their use of BCTs is restricted by a lack of capability, due to limited training. Furthermore, the opportunity to effectively utilise BCTs is restricted by organisational environment, resources and effective clinical supervision.

**Conclusions:**

Improving physiotherapists' capabilities by enhancing BCT training and psychology led clinical supervision is required. Furthermore, organisational change is recommended to create the opportunity for clinicians to effectively utilise BCTs. Specifically, organisations should devote necessary resources, backed by effective implementation strategies, to enhance such opportunity.

## 1 Background

The role of a physiotherapist is to ‘help people affected by injury, illness or disability through movement and exercise, manual therapy, education and advice’ [1]. To achieve desired clinical outcomes, physiotherapists often advise several techniques which go beyond clinic‐based appointments. These require regular engagement from patients and may include changes to self‐management behaviours such as exercise [2]. Unfortunately, multiple studies have found that patient adherence to such recommendations is inconsistent and influenced by multiple factors, including the extent of knowledge held by healthcare providers regarding how to support patients to maintain adherence [3]. With an ageing population and the incidence of long‐term conditions increasing, the problem of adherence to treatment is greater than ever [4].

Adherence is defined as ‘the extent to which a person's behaviour corresponds with agreed recommendations from a healthcare provider’ [5] and is an important predictor of positive patient outcomes such as reduced pain, greater mobility, and independence [6, 7]. Within physiotherapy, non‐adherence to treatment has been found to be as high as 65%; this can have a detrimental impact across a range of individual and service level outcomes including physical function, pain, avoidable morbidity and resource utilisation [8, 9, 10]. It is therefore a significant challenge for clinicians, patients and the wider healthcare system which requires further attention.

A range of biopsychosocial factors have been proposed to explain the lack of adherence found in physiotherapy practice, including the patient's diagnosis, symptomology, gender, age and level of motivation [11, 12, 13]. Identifying barriers to adherence early in the patient's care pathway can enable physiotherapists to work alongside patients to mitigate these barriers, facilitating the necessary behavioural changes [14]. However, despite such evidence outlining factors influencing patient adherence, the problem persists. Research is lacking to explain this from the perspectives of physiotherapists, including how equipped they feel to change such patient behaviour.

Adherence to clinical recommendations is a health behaviour and therefore strategies aiming to address non‐adherence may be informed by behaviour change theory. The Behaviour Change Technique (BCT) taxonomy [15] consists of 93 behaviour change techniques which can be applied by clinicians to facilitate behaviour change and improve self‐efficacy. As such, BCTs have the potential to enable greater adherence, leading to improved outcomes including enhanced self‐management skills and recovery potential [16, 17]. Examples of BCTs include goal setting, habit formation, social support and information about health consequences.

Evidence has demonstrated that the use of BCTs can be an effective strategy for physiotherapists to improve adherence to treatment in patients with musculoskeletal conditions [17, 18]. Bassett [19] suggests that behaviour change strategies can help patients integrate physiotherapy interventions into their everyday lives, thus mitigating problems with adherence and overcoming the intention‐behaviour gap. Despite evidence demonstrating the effectiveness of BCTs [20], and recommendations from the National Institute for Health and Care Excellence to embed BCTs in healthcare where appropriate [21], there are concerns they remain under‐utilised in clinical practice. Studies have demonstrated that physiotherapists only use a small number of BCTs when working with patients [22], with insufficient experience and a lack of training resulting in low self‐confidence and lack of awareness of BCTs being suggested as reasons for this [23]. Holopainen et al. [24] highlighted that physiotherapists do not feel adequately trained to apply psychological theories and techniques within their practice, which may go some way to explain their limited use of BCTs. However, this systematic review focused on a range of psychological interventions rather than BCTs specifically and so these warrant more focused investigation. Furthermore, several studies exploring physiotherapists use of psychological techniques have applied quantitative methodology, such as self‐report questionnaires [25, 26], which provide limited insight into the experiences and perceptions of physiotherapists regarding the issue.

Understanding of the barriers and facilitators perceived by physiotherapists to the use of BCTs within clinical practice is essential to develop interventions to address the challenges identified. This study, therefore, aimed to understand the ways in which physiotherapists use BCTs in clinical settings, and barriers and facilitators to implementing them.

## 2 Methods

### 2.1 Design

A qualitative, semi‐structured interview study was utilised to elicit beliefs about perceived barriers and facilitators to BCT use and to identify areas for change [27, 28]. Qualitative approaches explore real‐world subject matter, allowing researchers to benefit from rich data and insights from participants which support contextual understanding, grounded in human experience [29]. The epistemological position within the research study was one of social constructivism. The researcher was not seeking objective fact, but to enhance knowledge and develop an understanding of the subjective perceptions of physiotherapists’ regarding the use of BCTs in clinical practice.

### 2.2 Participants

Convenience sampling was applied, with advertisements shared at a higher education institution based in the north‐west of England and a community based musculoskeletal (MSK) and chronic pain care provider from April–July 2023, asking participants meeting the inclusion criteria to volunteer for the study. Purposive sampling was also used, with the researcher contacting physiotherapists within a community chronic pain service who it was believed could contribute useful knowledge regarding the subject, based on their clinical experience. The use of these two sampling techniques helped researchers to recruit physiotherapists with a range of experience, potentially enhancing the applicability of findings. A sample size of 14 participants was determined based on samples used elsewhere in the literature [30] and also considered the scope of the research, participant requirements and agreements with gatekeepers [31]. The inclusion criteria were: Over 18 years old, practicing physiotherapists registered with the Health and Care Professions Council, and at least 12 months post‐qualifying experience. This ensured participants had enough experience to contribute useful insights during interviews, thereby enhancing data quality. Participants were excluded if they were not working in a clinical setting, could not understand or converse in English, or could not complete interviews either face to face or using Microsoft Teams. Friends and family of the researchers were not eligible to participate.

### 2.3 Procedure

Gatekeepers were fully informed regarding the nature of the research and selected based on their access to eligible participants. They assisted with recruitment by circulating an interview invitation via email. Eligible participants were asked to self‐identify by contacting the researcher via email to express interest. They were then provided with a participation information sheet detailing the study in full and asked to familiarise themselves with the requirements of the study, confirming their willingness to participate within 48 h.

All interviews were conducted using Microsoft Teams recording and transcription feature. Participants were asked to complete a short demographics questionnaire and return this before the interview. Participants were encouraged to ask any questions to ensure they were fully informed of the aims of the study, participant requirements and data protection safeguards. When participants were satisfied, audio consent was recorded before the interviews which is an efficient and secure method of obtaining consent [32]. Completed interview recordings and transcriptions were stored in a password‐protected folder on the university One Drive storage facility, in line with GDPR.

Semi structured interviews were conducted which allowed the researcher flexibility to pursue topics as they arose, obtaining rich data and subjective insights from participants [33, 34]. The interviewer was a MSc Health Psychology student who was also an Occupational Therapist and Clinical Lead working in chronic pain management. An interview schedule was developed with the aim of building trust and rapport, encouraging rich and usable data from participants and providing a framework for consistency across multiple interviews. Open questions were utilised and the interviews started with a broad opening question, aimed at putting participants at ease. Further questioning techniques were applied throughout, including example, prompting and summary questions [35]. Probing questions also enabled the researcher to seek clarity, detail, and enhance data credibility [36]. Interviews lasted between 19 and 44 min, based upon the engagement of the participants. However, all interviews followed the full interview schedule, displayed in Table 1.

**Table 1:** Interview schedule.

1. Are you able to talk me through a typical patient appointment?
2. What are some of the challenges you experience when working with patients?
3. What are some common psychosocial factors you encounter during your assessment and treatment planning and how might you mitigate these? a. Do you have any specific examples of strategies, techniques, questions or prompts that you use?
4. Tell me how you would help patients overcome barriers to adherence with treatment plans? a. What are some examples of strategies, techniques, questions or prompts that you use?
5. What is your understanding of Behaviour Change Techniques (BCTS)? E.g., goal setting, social support, reward, habit formation, self‐monitoring, reducing negative emotions, framing/reframing
6. How confident do you feel applying BCTs, and could you provide any examples you use in clinical practice?
7. Are there any specific barriers that restrict your use of BCTs with patients? E.g., confidence, time, training
8. What, if anything, could be done to equip clinicians with the skills and confidence to use BCTs regularly during appointments?
9. Please consider this case study and how you would respond: An elderly patient attends a second appointment with you and is reporting low levels of motivation and self‐confidence to engage with their treatment plan. This is resulting in a lack of adherence and clinical improvements. How might you work with them to improve their adherence?
10. In conclusion, how would you sum up your awareness and use of BCTs within clinical appointments?

### 2.4 Data Analysis

Data was analysed using reflexive thematic analysis, a theoretically flexible method for identifying, analysing and interpreting patterns of meaning within qualitative data [37]. Reflexive thematic analysis involves extensive analysis of the data from the researcher and follows six distinct steps as set out by Braun and Clarke [38]; familiarisation; coding; generating initial themes; developing and reviewing themes; refining, defining and naming themes; and writing up. In the present study, familiarisation involved immersion with the data by watching the video interviews, checking the accuracy of transcripts and rereading multiple times while logging initial considerations. Similarities between transcripts were noted at this stage. When transcribing, data was also cleaned by removing repetition, hesitation and adding punctuation as described by Braun and Clarke [39]. During coding, the first author systematically worked through the full data set and attached code labels to segments of the data which were potentially relevant to the research question (see Appendix A).

A semantic approach was applied to coding, to present the data as communicated by physiotherapists, thereby explicitly understanding their views of barriers and facilitators to the use of BCTs. This was deemed appropriate as the research was focused on the specific topic of BCTs and the practical, everyday use of these techniques rather than seeking to identify hidden meaning, emotion or underlying ideologies which may have been contributing to the data [40]. The process of theme generation was facilitated by the constructivist approach of the study. This ensured that the researcher captured not only the participants’ experiences, but also the context from which these were drawn. For example, consideration of the working environment and organisational nuances. When generating initial themes, potentially connected codes were clustered into candidate themes to explore their meaning in relation to the research question. An inductive approach was taken to initial theme identification as the research was exploratory, seeking patterns which would enhance understanding of physiotherapists’ use of BCTs. Following this, themes were developed and reviewed by the researcher which involved reorganising and merging several code labels and candidate themes (see Appendix B). During this stage all code labels were revisited, ensuring they were backed by meaningful data and presented a coherent pattern with relevance to both the proposed theme and research question. This was discussed with the research team to come to final agreement of the themes. When this process was complete, the final themes were defined and named to reflect the data within them. An audit trail was maintained throughout to document analytical decisions.

### 2.5 Methods to Ensure Rigor

The researcher has aimed to ensure rigor throughout the research process. Dependability has been demonstrated by providing transparency and detail regarding the methods of data collection, analysis and interpretation. Appropriate sampling choices also helped to enhance the rigor of the study [41]. To ensure transferability the participant voice is represented alongside detailed contextual information is presented in addition to an information rich sample of participants. It should be noted that the researcher is a qualified Occupational Therapist working within a chronic pain management service and was therefore able to relate to physiotherapists from a chronic pain background more so than from acute or musculoskeletal physiotherapy backgrounds. During initial interviews this resulted in a more conversational approach which could have been interpreted as the researcher leading aspects of discussions. This was noted by the researcher early in the interview process following a period of reflective journalling. In subsequent interviews the researcher ensured they followed the discussion by employing techniques such as paraphrasing and clarifying. The researcher also manages and supervises physiotherapists within chronic pain management services and has noted that some clinicians lack in BCT skills. This influenced their decision to pursue this study and may also have resulted in some underlying assumptions as to why such skills may be lacking. In an attempt to mitigate this, semantic coding and multiple reviews of data extracts ensured that the perspectives of the physiotherapists were provided, rather than those of the researcher.

### 2.6 Ethical Considerations

Ethical approval was granted by the by the Manchester Metropolitan University Research Ethics Committee (Ref: 53858). All participants were provided with detailed study information and provided informed consent to participate. Upon completion of interview transcription all recordings were destroyed and personal data (such as names, places discussed in interviews) replaced with pseudonyms. Data was anonymised using a unique pseudonym, kept confidentially and only used for the stated aims of the research.

## 3 Results

The final sample (*N* = 14; *F* = 8, *M* = 6) consisted of chartered physiotherapists with an age range of 24‐58 (*M* = 38.14, SD = 10.19). Participants practiced in a range of settings including hospitals, clinics, educational settings, and private practices. A detailed summary of participant demographics is shown in Table 2.

**Table 2:** Participant demographics.

Name and sex	Age	Setting of practice	Years of clinical experience	Received formal training in behaviour change techniques (Y/N)
Terry (M)	45	Private	4 or more	No
Bill (M)	24	Private Education	3	No
Timothy (M)	43	Education	4 or more	Yes—As part of chronic pain training
Darren (M)	28	Clinics	4 or more	No
Alan (M)	34	Hospital Clinics	4 or more	No
Carol (F)	58	Clinics	4 or more	Yes—CBT, ACT, Motivational Interviewing
Katie (F)	38	Clinics	4 or more	No
Cathy (F)	29	Private	4 or more	No
Diana (F)	47	Clinics	4 or more	No
Ian (M)	50	Hospital	4 or more	No
Charlotte (F)	41	Clinics	4 or more	No
Greta (F)	25	Hospital	1	Yes—Undergraduate programme
Yasmin (F)	42	Private	4 or more	No
Elouise (F)	30	Private	4 or more	No

Three themes were identified using the reflexive thematic analysis: (1) A Lack of Training and Support; (2) Organisational Culture and Practices; (3) Individual Differences During the Consultation, which also had two subthemes: (i) Patient Attitudes and Expectations, (ii) Clinician Attitudes and Approach. Together, these themes provide insights into the barriers and facilitators perceived by physiotherapists to the use of BCTs to improve patient adherence.

### 3.1 A Lack of Training and Support

This theme highlights that a lack of training throughout both physiotherapy undergraduate programmes and continuous professional development (CPD) leads to a lack of awareness of BCTs and their scope and a lack of confidence utilising them in practice. The theme reflects a lack of capability caused by limited knowledge and skills:

So I found it's something [BCT training] that I did very little of as a physio student or in my general initial rotations within a hospital. It wasn't something that was strongly reinforced or taught.

(Alan)

The suggestion that physiotherapy undergraduate training programmes did not include BCT skill development was common in the data. Without this foundation, clinicians report a genuine lack of awareness of BCTs. As Charlotte described ‘*you don't know what you don't know.’* Indeed, during multiple interviews the researcher was required to clarify the nature of BCTs, providing examples to facilitate further discussion. This lack of awareness was consistent:

I'm sure there are more behaviour change techniques out there than what we are using, which would be really beneficial to learn about.

(Charlotte)

With the majority of participants, this lack of awareness extended to the BCT Taxonomy [15]. Interestingly Charlotte was the only participant who described definitive awareness of the BCT Taxonomy. However, she reported:

I think the only the only thing that I found with the taxonomy […] it was quite overwhelming. I remember there was lots on it and it was a little bit like, oh, I don't know where to start.

(Charlotte)

The lack of awareness of the BCT taxonomy and lack of clarity regarding its use in practice was revealing, and has recently been described elsewhere in the literature [42]. Improving clinician resources, such as step‐by‐step guides to implementing BCTs, may help to address such capability gaps. Bill described the perceived benefits of a validated framework to facilitate physiotherapists’ use of BCTs. Despite this, he was unaware that the BCT Taxonomy already exists:

And maybe [it would be beneficial to have] an encyclopaedia of behaviour changes that you can reference back to and say, OK, right. If I've got someone who's presenting with these beliefs […] I can look at different ways to challenge that belief.

(Bill)

When considering the research question, this reflects that physiotherapists have a limited understanding of the use of BCTs to improve patient adherence, despite evidence of their effectiveness [18]. They also appear to lack awareness of the theoretical underpinnings and frameworks of BCTs such as the BCT Taxonomy. The perceived lack of training in BCTs inevitably impacts clinician confidence:

No, I would say I am not confident at all because I've never had any formal training on behaviour change or behaviour change techniques.

(Terry)

This theme also captures potential facilitators towards improved BCT use, as described by participants, namely improved CPD, clinical supervision and multidisciplinary team (MDT) working:

You can learn the techniques, but it's actually applying them and in an environment where you feel safe to do so as well and have that adequate supervision to discuss when things don't go to plan.

(Carol)

As expressed here by Carol, having a safe and supportive environment for skill development, backed by effective clinical supervision, can play a crucial role in building clinician confidence when implementing new practices [43]. Therefore, even when the issue of capability has been addressed, the opportunity to utilise BCTs must be considered to ensure clinicians feel safe and supported.

### 3.2 Organisational Culture and Practices

This theme reflects the notion that the organisation within which the physiotherapist is working can either facilitate or restrict the opportunity to utilise BCTs. A pattern noted by the researcher within the data was the contrast drawn between physiotherapists working in acute musculoskeletal settings and those working in chronic pain clinics. This is reflected by Timothy:

I think part of the education around it [BCTs] in Physio tends to be almost hung on chronic pain management and actually what people don't understand is that it applies to anybody […] this just doesn't apply to chronic pain patients, this applies to literally everybody and it's so important.

(Timothy)

The suggestion that physiotherapists working in chronic pain are more adept at engaging patients with BCT discussions was prevalent across interviews. Participants suggested that this may be attributable to the multi‐disciplinary, biopsychosocial approach commonly used in chronic pain treatment, compared to MSK physiotherapy settings which may focus more on acute physical dysfunction:

MSK physiotherapists follow a medical approach and are looking for the diagnostics and then the treatment and you know what, I feel there's a big gap. There is a big gap with physiotherapists in terms of the biopsychosocial approach.

(Yasmin)

I'd say more of it [BCT awareness] has been supported at work by people in our pain management teams and the clinical psychologists rather than being directed by physios.

(Katie)

Here, Katie links together both the present theme and ‘A Lack of Training and Support’ by highlighting that much of her capability has been developed via multidisciplinary working within pain management settings and from colleagues with specialist training, suggesting a role for psychologists in providing CPD and supervision.

Another contrast was drawn between National Health Service (NHS) and private organisations relating to the lack of psychosocial input within NHS settings:

The patients themselves will tell you […] they've been to an NHS physio and they've just been given a sheet of exercises and told to go away and never really felt like they'd had a full assessment […] and that's why so many will come into the private practice because they didn't feel like they were getting what they needed from the NHS.

(Cathy)

In this discussion regarding psychosocial patient assessment, Cathy links to a notion of ‘exercise prescription’ which was found elsewhere in the data. This suggests that some physiotherapists provide exercises to patients but do not always reinforce this intervention with effective BCTs to help patients integrate them into their routine. A common explanation for such practices within the NHS was the lack of time in consultations and lack of opportunity to develop BCT skills:

And not to shit on the NHS, but I think they are put under so much pressure that they don't have the time or opportunity to work on that skill [BCT use], especially at a junior level.

(Bill)

A similar service‐level issue was raised by Katie, the lack of follow up appointments to review patients progress with potential BCT interventions:

I'm often not seeing patients for follow ups and it might just be a one off […] consultation and then a review to discuss a diagnostic result or something.

(Katie)

Bill and Katie highlight issues relating to the length or frequency of appointments which create a restrictive environment towards implementing BCT practices. Such organisational constraints are well established as barriers to implementation of evidence based practices [44, 45] and suggest a lack of opportunity to use BCTs in practice.

### 3.3 Individual Differences During the Consultation

This theme considers the unique nature of each consultation and the nuance in appointments created by the clinician and patient, with subthemes to reflect each individuals characteristics and how these shape the use of BCTs within appointments.

i. **Patient Attitudes and Expectations**
  This subtheme explores physiotherapists’ perceptions of their patients and how these may facilitate or restrict their own motivations to apply BCTs. Several participants highlighted that a patient*'*s readiness for change is a key factor in this:
  Maybe the more stressed or overworked patient may be less receptive to that kind of advice or behavioural change […] you get to know them patients quite quickly that perhaps this kind of intervention is not going to be successful for them, particularly in the short term.
  (Alan)
  Here, Alan explains that the characteristics of the patient and their presentation may impact the physiotherapists’ motivations, reducing their confidence in initiating BCT discussions due to the reduced likelihood that this would result in a positive outcome.
  Another factor reducing the likelihood of physiotherapists attempting to utilise BCTs was the expectations of patients. Participants commonly reported that patients who present expecting a diagnosis, cure, or hands‐on treatment such as manual therapy or injections are often not receptive to biopsychosocial discussions and BCTs:
  There is a part of the patient [group] who are just waiting for the golden pill and they don't wanna do something. They don't believe that exercises can help. They are more medicine focused, more passive treatment focused.
  (Elouise)
  The issue raised by Elouise is one that has also been reflected in nurses' experiences working in chronic pain settings [46]. This may be addressed by educating patients regarding long‐term self‐management of their condition, a common approach within chronic pain settings [47], linking back to the ‘Organisational Culture and Practices’ theme. Indeed, one BCT Taxonomy category which received recognition in several interviews was shaping knowledge [48]. Multiple physiotherapists spoke of the importance of patients understanding the reasons for their treatment plan to enhance adherence:
  A lot of patients have been given exercise sheets [without] being educated why it's important. They don't know the science behind why it's important to engage in them exercises.
  (Yasmin)
ii. **Clinician Attitudes and Approach**

This subtheme contains insights from physiotherapists on their own practice and how this may motivate BCT use. The clinician*'*s own approach to the appointment was perceived as key:

You really have to get the person on board and for them to see why a change would be beneficial for them to promote adherence and that can be a much more detailed conversation with quite some skill around it, as opposed to just giving out a sheet of exercise and expecting someone to get on with it.

(Charlotte)

Several physiotherapists were keen to stress that they approach patient appointments collaboratively and this was a facilitator to the use of BCTs to promote adherence, as described by Charlotte. However, some clinician attitudes and approaches present barriers to the use of BCTs:

I think it's [BCT use] part of our bread and butter. Basically, we may not have […] a fancy name for it, but it should be sort of like bread and butter.

(Ian)

Here, Ian suggests BCT use is considered a natural part of the clinicians practice, almost to the point of being dismissive of some techniques. Furthermore, BCT conversations may not be a clinician priority:

What is the purpose of that appointment and if the main purpose is to get to a sort of definitive answer in terms of diagnostics or where the patient ends up, then it's a conversation that is a nice‐to‐have rather than a definite necessity.

(Katie)

This suggests a lack of understanding regarding the scope and purpose of BCTs and how they may facilitate positive shared outcomes between patients and clinicians, further emphasising the need to improve clinician knowledge and capability.

## 4 Discussion

This study aimed to understand the ways in which physiotherapists use BCTs in clinical settings, and barriers and facilitators to implementing them. Fourteen participants engaged with semi‐structured interviews, with three themes identified in the data: (1) A Lack of Training and Support; (2) Organisational Culture and Practices; (3) Individual Differences During the Consultation, which had two subthemes: (i) Patient Attitudes and Expectations; (ii) Clinician Attitudes and Approach.

*A Lack of Training and Support* highlighted that physiotherapists do not feel they have adequate training in BCTs to regularly apply them in practice, in keeping with previous literature describing physiotherapists’ lack of training in wider psychological interventions [49]. The lack of training described by participants led to several barriers: A lack of confidence, experience and awareness of both BCTs generally and the BCT Taxonomy. Such barriers have been described previously [23, 42], however this study offers insights from the perspectives of physiotherapists regarding these issues. Three participants reported that they had prior BCT training, and they appeared to demonstrate deeper understanding of the use of techniques and associated benefits. This suggests a role for more widespread training for physiotherapists. This theme also captured the perceived value of MDT working and clinical supervision as facilitators to improving BCT use. This emphasises the importance of a robust supervision framework to build clinicians’ confidence when adapting to new clinical practices, particularly in relation to implementation of BCTs.

The *Organisational Culture and Practices* theme highlighted how physiotherapists’ practice is influenced by the organisation they work in. Central to this theme was the distinction between physiotherapists working in chronic pain settings and those working in acute musculoskeletal care. A multi‐disciplinary approach, underpinned by a biopsychosocial framework and utilising BCTs such as shaping knowledge is now common in chronic pain settings and the benefits of this have been documented elsewhere [49, 50]. However, several participants felt that such an approach was lacking in acute musculoskeletal settings, despite their willingness to practice in such a way. This was attributed to the enhanced level of CPD and psychology‐led supervision available within pain management MDTs. A further organisational issue was the pressures of working in NHS settings. Restrictions on appointment times and a lack of follow‐up appointments were highlighted as barriers to BCT use. Previous literature has illustrated that implementation of new clinical practices can be adversely affected by time constraints, a lack of resources and organisational support [51], demonstrating a need for systemic change and support to enhance physiotherapy practice and, by extension, patient outcomes.

The *Individual Differences During the Consultation* theme contained two subthemes which described how the attitudes of both patients and clinicians may influence physiotherapists’ motivation to apply BCTs. The patients’ readiness for change and expectations of the appointment were perceived as key. Interestingly, participants described very little control or influence over such patient attitudes, despite the fact that they may be adapted by effective use of BCTs, such as shaping knowledge [48]. The need for patients to understand their condition and link this to the treatment plan has previously been reported by Miller [52], who found that enhancing health literacy of patients can improve adherence. Further, participants felt that they used BCTs naturally without specific training and others did not feel they were always a priority during appointments. This is consistent with previous studies which have highlighted a lack of consideration of psychosocial influences during physiotherapy appointments [24], and highlights the value of recognising the unique requirements of each patient from one consultation to the next.

The data is reflective of an underlying motivation to utilise BCTs. However, actual implementation in practice is hindered by a lack of capability due to limited knowledge and skills, and a lack of opportunity due to organisational constraints such as time and resources. Enhancing the provision of BCT‐focused CPD and psychology‐led clinical supervision to replicate chronic pain services would help to strengthen musculoskeletal physiotherapy practice and improve adherence to NICE guidance (2007) on use of BCTs in healthcare. This includes enhancing clinician confidence, experience, and awareness of BCTs, along with managing patient expectations, shaping patient knowledge and dealing with patient resistance to behaviour change, all of which will result in greater application of BCTs in practice. The data suggests that physiotherapists working in fast‐paced clinics do not feel adequately supported to widely adopt new practices, such as BCTs, due to restrictions on time and frequency of appointments. Environmental restructuring can aid with implementation of such practices as described by McArthur et al. [51]. Examples of such changes include allocating clinicians sufficient time for training and supervision, extending the length and frequency of patient appointments and improving resources to facilitate the use of BCTs in clinical settings.

### 4.1 Implications and Future Research

The findings suggest a range of potential interventions that influence capability (BCT training and skills development), opportunity (addressing structural/organisation barriers to BCT implementation) and motivation (e.g., improving confidence in BCT use), in line with the COM‐B model of behaviour change [48]. The COM‐B model is widely used to identify and understand determinants of **b** ehaviour through ***c** apabilities* (capacity to engage in behaviour), ***o** pportunity* (environmental factors that influence behaviours) and ***m** otivation* (the willingness to change), factors known to influence an individual's capacity to adopt new behaviours. This has the advantage of enabling the development of a theoretically driven individually tailored intervention to support and empower physiotherapists to develop their skills and implement BCTs in practice, where appropriate. This study therefore provides the foundation for developing theory and evidence‐based interventions to improve physiotherapist practice, and outcomes for their patients. Table 3 summarises key recommendations from this study:

**Table 3:** Recommendations.

Recommendation	Rationale and impact
Improvements should be made to training in the use of BCTs.	To improve physiotherapists' knowledge, skills and confidence regarding the use of BCTs.
Improved utilisation of BCTTv1	To improve the application and impact of BCTs in clinical settings.
Development of formal training resources	
Directed CPD	
Enhanced clinical supervision for all clinicians delivering BCTs.	To enable physiotherapists to feel safe, supported, and confident in the delivery of BCTs and psychologically informed practices.
	To ensure BCT‐focused clinical supervision is provided by appropriately qualified psychologists.
	To improve physiotherapists confidence in addressing psychosocial issues.
Organisational changes to enhance the opportunity for physiotherapists to apply BCTs effectively.	To enable the development of service‐specific implementation strategies which will lead to improved use of BCTs.
Improved resources	To create the opportunity and environment for clinicians to effectively utilise BCTs.
Effective implementation practices	
‘Behaviour Change Champions’ to drive implementation	To help to embed BCT use into practice.

Findings from this study suggest that improving physiotherapists’ knowledge and skills regarding the use of BCTs is essential to improving current practice, enhancing their capability. Central to this is improving the provision of BCT focused continuous professional development (CPD). The Health and Care Professions Council (HCPC) state that ‘You must only delegate work to someone who has the knowledge, skills and experience needed to carry it out safely and effectively’ [53]. Some physiotherapists currently feel this is not the case with BCTs. CPD, such as education and training, can be an effective strategy for supplementing knowledge and skill gaps, ensuring clinicians are meeting recommendations from regulatory bodies such as the HCPC and improving the implementation of evidence into practice [54]. Effective training for clinicians can be offered in several formats including self‐directed learning, observations of practice and formal and informal education [55, 56]. Physiotherapists should undertake CPD focused on improving knowledge and application of the Behaviour Change Technique Taxonomy Version 1 (BCTTv1) [15], as this will equip them with a theoretical framework from which behaviour change interventions can be planned and delivered to patients. The Medical Research Council states that behaviour change theory should be applied when developing interventions which seek to promote behaviour change as this results in better outcomes than nontheoretical approaches [57]. It has been suggested that without robust theoretical underpinning, outcomes would be largely driven by chance rather than method [58]. Unfortunately, the prevalence of behaviour change interventions that do not use a theoretical framework remains high [59], suggesting that clinicians are not utilising evidence‐based tools such as the BCTTv1, as was reflected in this study. The BCTTv1 was developed, in part, to address this issue and this recommendation seeks to promote its use to enhance the psychological capability of physiotherapists. Unfortunately, there is a lack of formal training regarding use of BCTs which is directed at clinicians. Therefore, a further recommendation of this study is for the development of training programmes and resources, aimed specifically at clinical practitioners, in the application of BCTs in clinical settings.

The importance of effective clinical supervision has been noted as a pattern throughout the data. Some participants suggested that they would not feel safe or confident utilising BCTs without appropriate supervision and so may avoid doing so. Therefore, a further recommendation is to enhance the provision of psychology led clinical supervision within physiotherapy settings to further address psychological capability issues, improving confidence and subsequent motivation to apply BCTs. Current clinical supervision recommendations from the Health and Care Professions Council (HCPC) state that when providing supervision, ‘You must keep within your scope of practice by only practising in the areas that you have appropriate knowledge, skills and experience for’ [53]. This suggests that supervision around BCTs should be provided by a clinician with experience and training in the techniques, such as psychologists. This is rarely the case in acute MSK clinics, where supervision is typically led by more experienced physiotherapists [60]. While this remains central to professional standards and should not be discounted, the addition of psychology led supervision may enable physiotherapists to enhance their practice, improving their use of BCTs and consideration of psychosocial factors which may influence a patients’ presentation. Such psychosocial factors are now routinely embedded within MSK best practice guidelines and with evidence demonstrating limited confidence and consideration of these from physiotherapists [61, 62], the benefits of psychology led clinical supervision may extend beyond improving BCT use.

The recommendations noted above, to improve training and supervision with the intention of strengthening physiotherapists’ psychological capability, require some organisational flexibility and willingness to embrace new practices. Further organisational change is recommended to create the opportunity for clinicians to effectively utilise BCTs. Therefore, this paper recommends organisations devote necessary resources, backed by effective implementation strategies, to enhance such opportunity. The implementation of evidence‐based interventions, such as those included in the BCT Taxonomy, has numerous benefits, including improved patient outcomes, quality of care, consistency of practice and resource utilisation [63, 64]. Despite a wealth of evidence regarding the benefits of adopting evidence based practices [65, 66], implementation remains challenging in healthcare settings [67]. Several implementation strategies can be adopted by organisations to attempt to address such challenges and facilitate effective practice development. These include passive strategies such as educational materials, toolkits and visual aids and active strategies such as workshops, audit and reminders [68]. A combination of these approaches appears to be the most effective in implementing evidence‐based practices [69] and so this is recommended by this paper. However, the specific implementation details of this recommendation may differ between organisations depending on the current environment. For example, some organisations may need to develop educational materials such as prompts and step‐by‐step guides to utilising BCTs to make their application more efficient and accessible in practice. Other organisations may need to focus on improving protocols to allow additional time during appointments. The value of leadership and use of ‘champions’ is also recommended elsewhere in implementation science research [51]. Therefore, this paper also recommends that services appoint ‘Behaviour Change Champions’ to conduct audit of organisational needs and to oversee the development of implementation strategies which will lead to improved use of BCTs in clinical settings. Behaviour Change Champions may come from the existing workforce and be a clinician who recognises and applies BCTs in their own practice. They would play a valuable role in promoting the wider application of BCTs by driving organisational change using the implementation strategies noted above. They may also ensure their presence in clinical environments such as MDT meetings, reflective practice and complex case discussions to help to embed BCT use into practice. Such approaches, including the use of clinical case studies, were recognised as valuable, but currently lacking, by participants in this study.

### 4.2 Strengths and Limitations

This study was novel in its specific focus on BCTs and physiotherapists’ perceptions of the barriers and facilitators to their use. It has therefore provided new insights and a foundation for consideration of interventions aimed at improving physiotherapists knowledge and confidence of BCTs. One such insight is the perceived benefit of chronic pain practices when compared with acute musculoskeletal settings.

There are some limitations to note for the current study. Firstly, the content of undergraduate and CPD training available and undertaken by participants was not verified. Therefore, any recommendations regarding improved training in BCTs must be considered in the context of what is currently provided within educational programmes. Similarly, this study did not record the location of each participants undergraduate training, and it is likely that there is variation between institutions, particularly in diverse geographical locations. For example, one participant trained in the Netherlands and explained that this programme did contain elements of BCT training. Further research may account for such limitations, enabling results to be more transferrable and allowing exploration of best practices in physiotherapy education.

## 5 Conclusion

This is the first study to identify barriers and facilitators of BCT implementation in physiotherapy practice. This study provides comprehensive insight into why physiotherapists do/do not implement BCTs in their practice and represents the necessary first step in the development of interventions to improve this. Developing such an intervention that targets the salient issues presented here will have greater potential to change physiotherapist behaviour and improve outcomes for their patients. Key recommendations emerging from this study centre around improving BCT training and resources, enhancing supervision and support, addressing organisational barriers to BCT implementation, and future research to assess the impact of these strategies.

## Ethics Statement

Ethical approval was granted by the Manchester Metropolitan University Research Ethics Committee ref no: 53858.

## Consent

All participants were provided with detailed study information and provided informed consent to participate. Participant consent was audio recorded before the interviews. Consent for publication was obtained from all participants and recorded before interviews.

## Conflicts of Interest

The authors declare no conflicts of interest.

## Supporting information

Example Transcripts with Initial Code Labels.

Supporting Evidence for Formulation of Themes.

## Data Availability

The datasets generated and analysed during the current study are available at https://doi.org/10.23634/MMU.00634531.
