# Supplementary material for: Barriers and Facilitators Towards Physiotherapists' Use of Behaviour Change Techniques (BCTs) to Improve Patients' Adherence to Treatment
Source: J Eval Clin Pract. 2025 Dec 15;31(8):e70339. doi: 10.1111/jep.70339 (PMC12706131; doi:10.1111/jep.70339)
Supplement: Supplementary file 1 — Example Transcripts with Initial Code Labels. [file JEP-31-0-s002.docx]

**Appendix A**

**Example Transcripts with Initial Code Labels**

| Timothy 20:48  I think I've had a little bit of training on behaviour change just related to sort of chronic pain management and that was formalised in like a particular sort of training.  I went through a postgraduate course in Keele University over like it was over a few months actually, and that was really, really helpful.  But I think part of the education around it in Physio tends to be almost hung on like the chronic pain management and actually what people don't understand is that it applies to anybody.  And I think that that it would be a really good way of of trying to sort of just make clinicians aware of things as if to say this just doesn't apply to chronic pain patients, that applies to literally everybody and it's so important.  And I don't know how you could emphasize that enough to people, but actually that's one of the things that I can look back on 20 odd years of experience and actually realise it's the a lot of people call them the soft skills of getting people on board.  But that is the key to to having any successful outcome in any any rehab setting, whether it's elite sport, whether it's like a general population, elderly care or anything like that, you know.  Interviewer 21:56  Yeah, I think that's a really good point as well. I think this this type of stuff is quite embedded in chronic pain services, and there's a lot more psychosocial focus, isn't there and behaviour change focus.  But even for acute injury and things like that, we need to be really harnessing this I suppose?  Timothy 22:19  Yeah, yeah.  I mean, I actually think it's even more important than like your actual knowledge base, if I'm honest, because you're if you're not engaging with people in what they're interested in and you're not giving them tasks specific or task orientated rehab, you're onto an absolute nonstarter. | The need for improved training  The benefits of BCT focused CPD  Contrast between MSK and Chronic Pain practices  The importance of a collaborative approach to behaviour change  The need to adapt practices to unique patient needs |
| --- | --- |

**Charlotte**

| Charlotte 30:51 And as I said earlier, is it aligned to their values? Can they see the benefit in the change? And so yeah, it's difficult because it depends a little bit on on what it is about. But I would want to explore that further, and I'd also want to explore further a little bit around sort of as I said, expectations around why they were coming to see us in the service because again, you know, if they if their expectations are around that we're gonna fix something for them.  But we're asking them to self manage and do behaviour change. Then they're two quite different things that that aren't going to meet.  Interviewer 31:25 Yeah sure  Charlotte 31:28 So you know, if we are asking them to do something, what are the barriers for them to do it? And again, you know it might be helpful to explore the barriers in more detail. What is stopping them from adhering to that treatment plan and is there something in particular around it? | The importance of value based goal setting  Patients expecting a fix or cure  Patients may not be ready for change  The importance of a collaborative approach to behaviour change |
| --- | --- |

**Katie**

| Interviewer 17:17  Do you use anything in terms of resources?  Do you give people activity or exercise sheets for example to record their own progress on that you know, provides feedback loops, those types of things?  Katie 17:32  Yeah. Sometimes. Yes.  And and things like uh, you know, looking at sort of adherence to things and trying to understand what what would work for them, sort of how you know recording things so that they can see that they've actually done things and and and kind of given that kind of positive reinforcement that they have made a change.  But again, it really depends on the individual and I think within my role I'm often not seeing patients for follow ups and it it might just be a sort of 1 off or maybe maybe 1 consultation and then a review to discuss a diagnostic result or something.  So I don't have. I don't have as much time with the individual and I don't have that kind of and they sort of repeated consultations where I can pick up on…  And you know, following things through and how did you find that?  And you know, do we need to change anything? And what would be helpful now?  And you know, so I don't have the luxury of that I think within my job.  Interviewer 18:32  Yeah. So that that time issue and that lack of follow up are real of barriers to effective or regular use of these techniques with that patient group aren't they? | Use of specific BCT – positive reinforcement, diaries  A lack of follow up appointments or long delays for these can affect adherence  A lack of time within consultations |
| --- | --- |
