# Supplementary material for: Barriers and Facilitators Towards Physiotherapists' Use of Behaviour Change Techniques (BCTs) to Improve Patients' Adherence to Treatment
Source: J Eval Clin Pract. 2025 Dec 15;31(8):e70339. doi: 10.1111/jep.70339 (PMC12706131; doi:10.1111/jep.70339)
Supplement: Supplementary file 2 — Supporting Evidence for Formulation of Themes. [file JEP-31-0-s001.docx]

| **A lack of training and support** | |
| --- | --- |
| Code | Example Extracts |
| The need for improved training  Feeling overwhelmed by BCTS  Clinician lacks confidence and experience  The value of clinical supervision or MDT working  A lack of awareness of BCTs  A lack of awareness of BCT Taxonomy  BCT knowledge acquisition to implementation gap | *“I found it's something [BCT training] that I did very little of as a physio student or in my general initial rotations within a hospital.*  *It wasn't something that was strongly reinforced or taught” (Alan)*  *“You know it's making sure you feel you feel safe. And I think maybe a lot of clinicians will feel . . . I'm not, I'm just not gonna go there really.” (Carol)*  *“I think like I say, peer supervision or group supervision and exploring changes in behaviour change techniques would be really helpful.” (Charlotte)*  *“I think the only thing that I found with the taxonomy . . .* *I remember there was lots on it and it was a little bit like, oh, I don't know where to start.” (Charlotte)*  *“I'm sure there are more behaviour change techniques out there than what we are using, which you know, would be really beneficial to learn about.” (Charlotte)*  *“you can learn the techniques, but it's actually applying them and in an environment where you feel safe to do so as well and have that adequate supervision to discuss when things don't go to plan.” (Carol)* |

**Appendix B**

**Supporting Evidence for Formulation of Themes**

| **Organisational culture and practices** | | | |
| --- | --- | --- | --- |
| Code | | Example Extracts | |
| Contrast between MSK and chronic pain practices  Contrast between NHS and private practices  Rigid practices and unwillingness to change  A lack of time in consultations  A lack of follow up appointments or long delays | | *“I think part of the education around it [BCTs] in Physio tends to be almost hung on like the chronic pain management and actually what people don't understand is that it applies to anybody. And I think that that it would be a really good way of trying to sort of just make clinicians aware of things as if to say this just doesn't apply to chronic pain patients, that applies to literally everybody and it's so important.” (Timothy)*  *“I'd say more of it [BCT awareness] has been supported at work by people in our pain management teams and the clinical psychologists rather than being directed by physios and or any other kind of MSK background.” (Katie)*  *“the the fact the NHS is obviously non-fee paying, perhaps the expectation is lower whereas there's a higher level or higher expectation for patients that are paying for a particular service privately so they do expect a more thorough maybe management or treatment plan.” (Alan)*  *“And I think it's one of those ones where it's like, you know, trying to train a an old dog to do a new trick. It's hard. It's bloody hard because they're stuck in their ways.” (Bill)*  *“if they're telling you a lot of information about their pain history, you can very quickly run out of time . . . and realise that you haven't really actually gone anywhere with them or offered them any sort of advice or guidance.” (Charlotte)*  *“I'm often not seeing patients for follow ups and it might just be a sort of one off or maybe one consultation and then a review to discuss a diagnostic result or something.*  *So I don't have, I don't have as much time with the individual and I don't have that . . . repeated consultations.” (Katie)* | |
| **Individual differences during the consultation** | | | |
| Code | Subordinate Theme | | Example Extracts |
| Patients may not be ready for change  Patients expecting hands on treatment  Behaviour change may not be the patients priority  Patients expecting a fix or cure  A lack knowledge regarding reasons for the treatment plan  Patients are fearful of exacerbating their condition | Patient attitudes and expectations | | *“If their expectations are around that we're gonna fix something for them but we're asking them to self-manage and do behaviour change, then they're two quite different things that that aren't going to meet.” (Charlotte)*  *“But you know, if they're not ready to change or ready to engage, then you know, you can use all the behaviour change you like, It's not gonna work.” (Darren)*  *“I think there's this perception of physios, like all you do is massage people better, but a lot of it is trying to empower them to be able to manage the condition themselves.” (Timothy)*  *“if you haven't got secure safe housing you you're not really in a position to change your behaviour . . . in the hierarchy of needs it is something that you know, has to be there.” (Carol)*  *“I found over the years that if they haven't got a full understanding of why you're asking them to do something and what their problem is . . . they often don't adhere to things.” (Timothy)*  *“So we get people that are afraid of doing certain things just because they the possibility that they make the situation worse.” (Ian)* |

| **Individual differences during the consultation** | | |
| --- | --- | --- |
| Code | Subordinate Theme | Example Extracts |
| Use of specific BCTs  The importance of value based goal setting  The use of a biopsychosocial approach  Clinician focus on fixing physical dysfunction  BCT use is considered a natural part of practice  BCT conversations are not a clinician priority  The importance of a collaborative approach to behaviour change | Clinician attitudes and approach | *“So small goals . . . I think it's quite well taught on physio programs. So we do focus . . . very much on goal orientation.” (Alan)*  *“Yeah, I mean, I think certainly as I've got more experience, I don't think you can actually separate the biological and the psychological element because everything is just so sort of interlinked.” (Timothy)*  *“I think I think it's [BCT use] part of our bread and butter. Basically, we may not have . . . well, it's sort of like a fancy name for it, but it should be sort of like bread and butter.” (Ian)*  *“What is the purpose of that appointment and if the main purpose is to get to a sort of definitive answer in terms of diagnostics or where the patient ends up, then it's sort of a conversation [regarding behaviour change] that is nice to have rather than a definite necessity.” (Katie)*  *“I think a lot of it is having that relationship with someone. I think that is key for behaviour change. If you don't have that trust between you and your patient, I don't think you've got any chance to change someone's behaviour.” (Diana)* |
